# Supplementary material for: Association between hypoglycemic agent use and the risk of occurrence of nonalcoholic fatty liver disease in patients with type 2 diabetes mellitus
Source: PLoS One. 2023 Nov 22;18(11):e0294423. doi: 10.1371/journal.pone.0294423 (PMC10664876; doi:10.1371/journal.pone.0294423)
Supplement: S1 Checklist — (DOCX) [file pone.0294423.s001.docx]

STROBE Statement—checklist of items that should be included in reports of observational studies

|  | Item No. | Recommendation | Page  No. | Relevant text from manuscript |
| --- | --- | --- | --- | --- |
| **Title and abstract** | 1 | (*a*) Indicate the study’s design with a commonly used term in the title or the abstract | 2 | This retrospective cohort study used data from … |
|  |  | (*b*) Provide in the abstract an informative and balanced summary of what was done and what was found | 2 | We aimed to compare the risk of occurrence of NAFLD with respect to the use of different hypoglycemic agents in patients with type 2 diabetes…Compared to DPP-4i, TZD use was associated with the decreased risk of NAFLD. |
| Introduction | | | |  |
| Background/rationale | 2 | Explain the scientific background and rationale for the investigation being reported | 3 | …potential effects of the drugs on patients without pre-existing NAFLD have been poorly understood. Several studies have examined whether TZD or SGLT-2i have a beneficial effect on preventing NAFLD and related outcomes, but their findings have been inconsistent. |
| Objectives | 3 | State specific objectives, including any prespecified hypotheses | 3-4 | Therefore, we aimed this study assess the risk of NAFLD occurrence with respect to the use of TZD and SGLT-2i, compared to dipeptidyl peptidase-4 inhibitors (DPP-4i) in patients with type 2 diabetes. |
| Methods | | | |  |
| Study design | 4 | Present key elements of study design early in the paper | 4 | We designed a retrospective cohort study to examine… |
| Setting | 5 | Describe the setting, locations, and relevant dates, including periods of recruitment, exposure, follow-up, and data collection | 4 | This study utilized data from the National Health Insurance Service-National Sample Cohort (NHIS-NSC) 2.0 database. The NHIS-NSC is an administrative health dataset established by the NHIS—a compulsory national health insurance service that provides coverage for all citizens in South Korea. |
| Participants | 6 | (*a*) *Cohort study*—Give the eligibility criteria, and the sources and methods of selection of participants. Describe methods of follow-up  *Case-control study*—Give the eligibility criteria, and the sources and methods of case ascertainment and control selection. Give the rationale for the choice of cases and controls  *Cross-sectional study*—Give the eligibility criteria, and the sources and methods of selection of participants | 5-7 | Patients newly diagnosed with type 2 diabetes mellitus between 2003 and 2019 were included in this study…  Individuals were followed up from the index date to the earliest date among the occurrence of NAFLD, discontinuation of the study drug, the date of death, or the end of the study period. |
|  |  | (*b*) *Cohort study*—For matched studies, give matching criteria and number of exposed and unexposed  *Case-control study*—For matched studies, give matching criteria and the number of controls per case | 7-8 | In the propensity score matching process, we controlled for potential factors that could impact the use of specific hypoglycemic drugs as well as the risk of NAFLD. Confounding variables included… |
| Variables | 7 | Clearly define all outcomes, exposures, predictors, potential confounders, and effect modifiers. Give diagnostic criteria, if applicable | 4-8 | The outcome of interest was NAFLD, which was defined based on claims that included ICD-10 codes of…  …prescription information was extracted based on the drug codes of ingredients for each class of hypoglycemic agents…  As comorbidities, the presence of dyslipidemia, hypertension, …, or chronic kidney disease were considered.  Concomitant medications were defined as medications that individuals take for at least one overlapping day within the follow-up period. |
| Data sources/ measurement | 8* | For each variable of interest, give sources of data and details of methods of assessment (measurement). Describe comparability of assessment methods if there is more than one group | 4-8 | (Described throughout the Methods section) |
| Bias | 9 | Describe any efforts to address potential sources of bias | 5 | The active comparator, new-user study design was applied to minimize the possible bias arising from the non-experimental study design…. |
| Study size | 10 | Explain how the study size was arrived at | 8-9 | A total of 105,061 patients were newly diagnosed with type 2 diabetes patients between 2003 and 2019. After excluding 39,837 participants with no history of study drug prescriptions, 65,224 individuals were finally included in the study… |

Continued on next page

| Quantitative variables | 11 | Explain how quantitative variables were handled in the analyses. If applicable, describe which groupings were chosen and why | 7-8 | Details on variables described in ‘Confounding variables’ |
| --- | --- | --- | --- | --- |
| Statistical methods | 12 | (*a*) Describe all statistical methods, including those used to control for confounding | 8-9 | Propensity score matching was performed within each cohort to minimize potential selection bias…  A Cox proportional hazards model was used to obtain… |
|  |  | (*b*) Describe any methods used to examine subgroups and interactions | 9 | We performed a subgroup analysis based on patients’ age, sex, BMI, year of index date, and ingredients of the hypoglycemic drugs. |
|  |  | (*c*) Explain how missing data were addressed | 10 | S2 Table - 2,232 of patients not included due to a missing data… |
|  |  | (*d*) *Cohort study*—If applicable, explain how loss to follow-up was addressed  *Case-control study*—If applicable, explain how matching of cases and controls was addressed  *Cross-sectional study*—If applicable, describe analytical methods taking account of sampling strategy | 7 | Individuals were followed up from the index date to the earliest date among the occurrence of NAFLD, discontinuation of the study drug, the date of death, or the end of the study period… |
|  |  | (*e*) Describe any sensitivity analyses | 9 | To evaluate the potential impact of a delayed NAFLD diagnosis following the cessation of drug exposure, we performed a sensitivity analysis… |
| Results | | | | |
| Participants | 13* | (a) Report numbers of individuals at each stage of study—eg numbers potentially eligible, examined for eligibility, confirmed eligible, included in the study, completing follow-up, and analysed | 9-10 | Figure 2, Figure 3 |
|  |  | (b) Give reasons for non-participation at each stage | 10 | Figure 2, Figure 3 |
|  |  | (c) Consider use of a flow diagram | 10 | Figure 2, Figure 3 |
| Descriptive data | 14* | (a) Give characteristics of study participants (eg demographic, clinical, social) and information on exposures and potential confounders | 10-12 | Table 2, Table 3 |
|  |  | (b) Indicate number of participants with missing data for each variable of interest | 10-12 | Table 2, Table 3 |
|  |  | (c) *Cohort study*—Summarise follow-up time (eg, average and total amount) | 13-14 | Table 4, Table 5 |
| Outcome data | 15* | *Cohort study*—Report numbers of outcome events or summary measures over time | 13-15 | Table 4, Table 5 |
|  |  | *Case-control study—*Report numbers in each exposure category, or summary measures of exposure | *NA* | *NA* |
|  |  | *Cross-sectional study—*Report numbers of outcome events or summary measures | *NA* | *NA* |
| Main results | 16 | (*a*) Give unadjusted estimates and, if applicable, confounder-adjusted estimates and their precision (eg, 95% confidence interval). Make clear which confounders were adjusted for and why they were included | 13-15 | Table 4, Table 5 |
|  |  | (*b*) Report category boundaries when continuous variables were categorized | 10-11 | Table 2 |
|  |  | (*c*) If relevant, consider translating estimates of relative risk into absolute risk for a meaningful time period | NA | NA |

Continued on next page

| Other analyses | 17 | Report other analyses done—eg analyses of subgroups and interactions, and sensitivity analyses | 14-15 | The comparison between DPP-4i and TZD in the subgroup analysis showed that…  Sensitivity analysis performed by setting the latency period to 30, 60, 90, and 120 days after drug discontinuation showed… |
| --- | --- | --- | --- | --- |
| Discussion | | | | |
| Key results | 18 | Summarise key results with reference to study objectives | 15 | Our results indicate that the use of TZD was associated with a significantly lower risk of NAFLD compared to the use of DPP-4i. A similar trend was observed when comparing SGLT-2i and DPP-4i, but this association was not statistically significant…. |
| Limitations | 19 | Discuss limitations of the study, taking into account sources of potential bias or imprecision. Discuss both direction and magnitude of any potential bias | 18-19 | Despite these strengths, this study has several limitations. Due to the nature of claims data, this study was unable to incorporate laboratory findings or detailed data on imaging or biopsy into identifying NAFLD… |
| Interpretation | 20 | Give a cautious overall interpretation of results considering objectives, limitations, multiplicity of analyses, results from similar studies, and other relevant evidence | 15-18 | The findings of the current study are consistent with those of previous retrospective studies... |
| Generalisability | 21 | Discuss the generalisability (external validity) of the study results | 18 | The results of this study gained more power and generalizability by utilizing a database that captured real-world clinical practice. |
| Other information | |  | | |
| Funding | 22 | Give the source of funding and the role of the funders for the present study and, if applicable, for the original study on which the present article is based |  | Funding information is entered in financial disclosure section of the submission system. |

*Give information separately for cases and controls in case-control studies and, if applicable, for exposed and unexposed groups in cohort and cross-sectional studies.

**Note:** An Explanation and Elaboration article discusses each checklist item and gives methodological background and published examples of transparent reporting. The STROBE checklist is best used in conjunction with this article (freely available on the Web sites of PLoS Medicine at http://www.plosmedicine.org/, Annals of Internal Medicine at http://www.annals.org/, and Epidemiology at http://www.epidem.com/). Information on the STROBE Initiative is available at www.strobe-statement.org.
