# Supplementary figures and images for: Association between hypoglycemic agent use and the risk of occurrence of nonalcoholic fatty liver disease in patients with type 2 diabetes mellitus
Source: PLoS One. 2023 Nov 22;18(11):e0294423. doi: 10.1371/journal.pone.0294423 (PMC10664876; doi:10.1371/journal.pone.0294423)

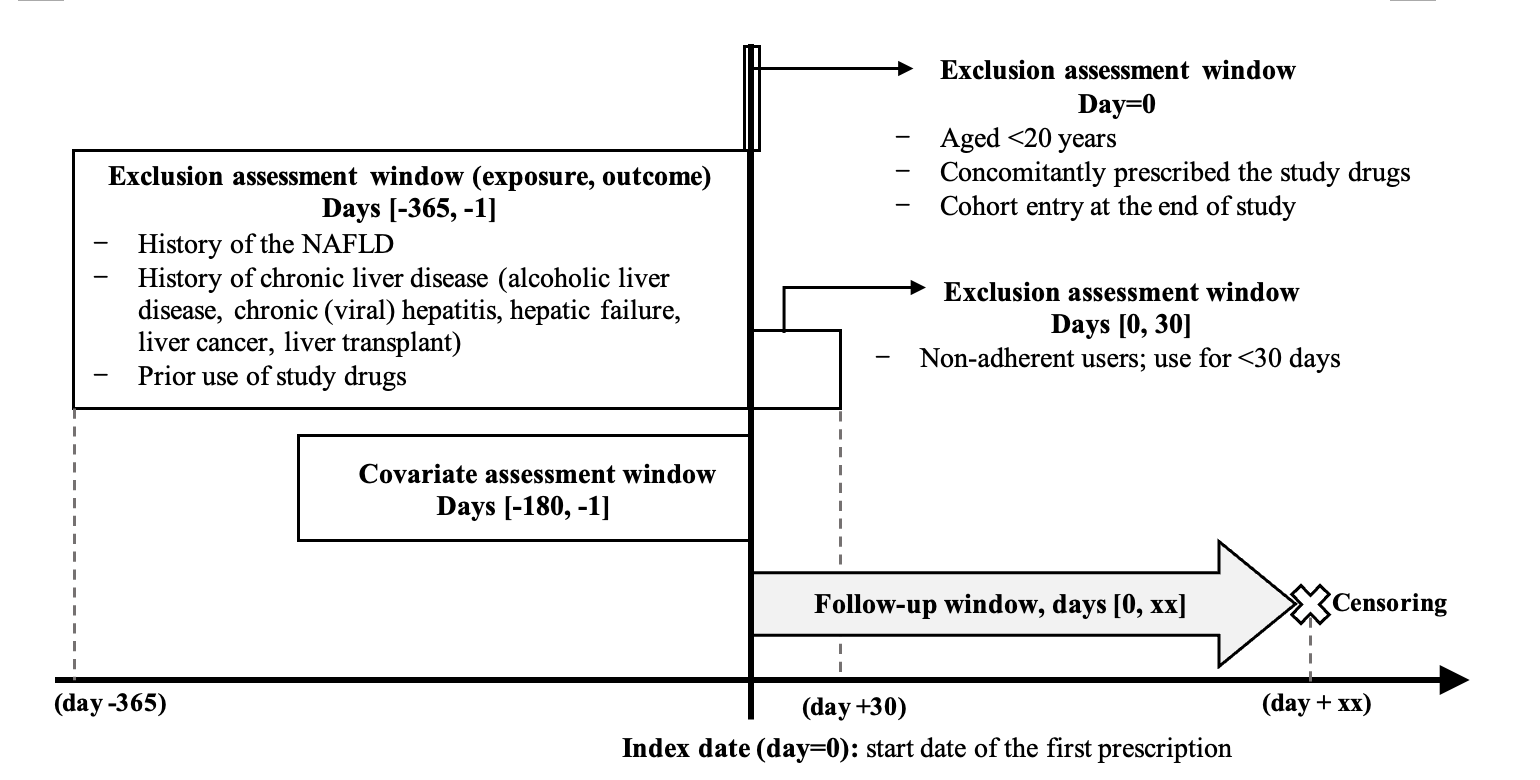

Supplement: S1 Fig — (TIF) [file pone.0294423.s002.tif]

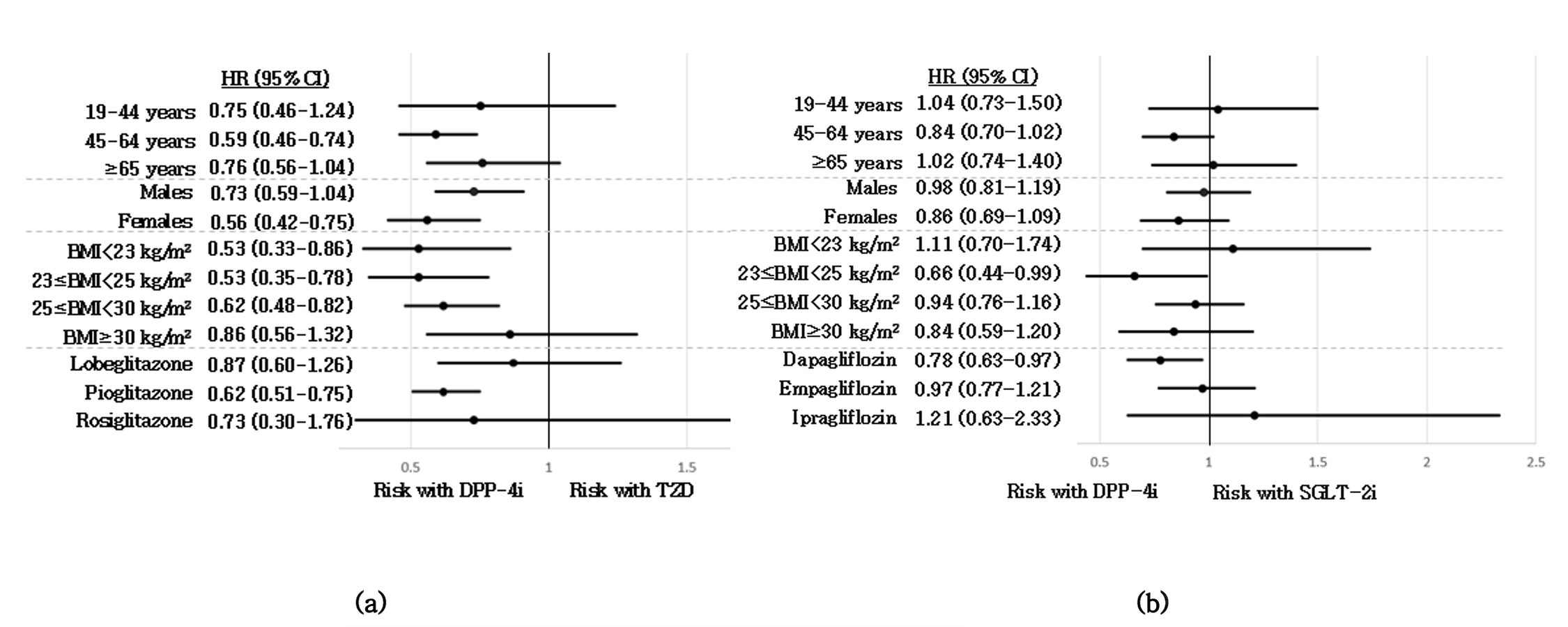

Supplement: S2 Fig — Subgroup analysis according to age, sex, body mass index, and ingredients (a) Cohort 1 (b) Cohort 2. (TIF) [file pone.0294423.s003.tif]
