# Supplementary material for: Association between hypoglycemic agent use and the risk of occurrence of nonalcoholic fatty liver disease in patients with type 2 diabetes mellitus
Source: PLoS One. 2023 Nov 22;18(11):e0294423. doi: 10.1371/journal.pone.0294423 (PMC10664876; doi:10.1371/journal.pone.0294423)
Supplement: S1 Table — (DOCX) [file pone.0294423.s004.docx]

|  | Disease(s) | | | ICD-10 codes |
| --- | --- | --- | --- | --- |
| **Study**  **Outcome** | Nonalcoholic fatty liver disease | | | K760 |
|  | Nonalcoholic steatohepatitis | | | K758 |
| **Chronic liver diseases** | Alcoholic liver disease | | | K70 |
|  | Toxic liver disease with chronic liver conditions | | Toxic liver disease with  chronic hepatitis | K713  K714  K715 |
|  |  |  | Toxic liver disease with  fibrosis and cirrhosis | K717 |
|  | Hepatic failure | | | K72 |
|  | Chronic hepatitis | | | K73 |
|  | Fibrosis and cirrhosis of liver | | | K74 |
|  | Malignant neoplasm  of liver | Malignant neoplasm of liver and intrahepatic bile ducts | | C22 |
|  |  | Secondary malignant neoplasm of liver and intrahepatic bile duct | | C787 |
|  | Liver transplant status | | | Z944 |
|  | Liver transplant failure and rejection | | | T864 |
|  | Chronic viral hepatitis | | | B18 |
|  | Acute delta-(super)infection in chronic hepatitis B | | | B170 |

Abbreviations: ICD-10, The 10^th^ revision of International Classification of Diseases
