# Supplementary material for: Association between hypoglycemic agent use and the risk of occurrence of nonalcoholic fatty liver disease in patients with type 2 diabetes mellitus
Source: PLoS One. 2023 Nov 22;18(11):e0294423. doi: 10.1371/journal.pone.0294423 (PMC10664876; doi:10.1371/journal.pone.0294423)
Supplement: S2 Table — (DOCX) [file pone.0294423.s005.docx]

|  | Total,  n | NAFLD,  n | Prevalence,  % | *p*-value ^a)^ |
| --- | --- | --- | --- | --- |
| Overall | 65,224 | 27,771 | 42.6 | - |
| Sex ^b)^ |  |  |  | 0.0029 |
| Males | 36,541 | 15,613 | 42.7 |  |
| Females | 26,451 | 11,617 | 43.9 |  |
| Age groups ^c)^ |  |  |  | <0.0001 |
| 19-44 years | 12,805 | 5,981 | 46.7 |  |
| 45-64 years | 36,526 | 15,875 | 43.5 |  |
| ≥65 years | 15,606 | 5,803 | 37.2 |  |
| BMI groups ^d)^ |  |  |  | <0.0001 |
| BMI<23 kg/m^2^ | 6,610 | 2,368 | 35.8 |  |
| 23≤BMI<25 kg/m^2^ | 7,907 | 3,331 | 42.1 |  |
| 25≤BMI<30 kg/m^2^ | 15,445 | 7,353 | 47.6 |  |
| BMI≥30 kg/m^2^ | 4,170 | 2,271 | 54.5 |  |
| Years |  |  |  | <0.0001 |
| 2003-2007 | 20,710 | 8,716 | 42.1 |  |
| 2008-2012 | 17,712 | 7,818 | 44.1 |  |
| 2013-2017 | 20,087 | 8,656 | 43.1 |  |
| 2018-2019 | 6,715 | 2,580 | 38.4 |  |

Abbreviations: NAFLD, nonalcoholic fatty liver disease; BMI, body mass index.

^a)^ *p*-value from chi-square test

^b)^ 2,232 of patients not included due to a missing data

^c)^ 287 of patients not included due to a missing data

^d)^ 31,092 of patients not included due to a missing data
