# Supplementary material for: Association between hypoglycemic agent use and the risk of occurrence of nonalcoholic fatty liver disease in patients with type 2 diabetes mellitus
Source: PLoS One. 2023 Nov 22;18(11):e0294423. doi: 10.1371/journal.pone.0294423 (PMC10664876; doi:10.1371/journal.pone.0294423)
Supplement: S3 Table — (DOCX) [file pone.0294423.s006.docx]

|  |  | Cohort 1 (DPP-4i vs. TZD) | | Cohort 2 (DPP-4i vs SGLT-2i) | |
| --- | --- | --- | --- | --- | --- |
|  |  | **Exposure** | **HR (95% CI)** | **Exposure** | **HR (95% CI)** |
| **Latency**  **period** | **Main analysis**  **(latency = 0 days)** | DPP-4i | 1.00 (ref) | DPP-4i | 1.00 (ref) |
|  |  | TZD | 0.66 (0.55-0.78) | SGLT-2i | 0.93 (0.80-1.08) |
|  | **latency = 30 days** | DPP-4i | 1.00 (ref) | DPP-4i | 1.00 (ref) |
|  |  | TZD | 0.68 (0.57-0.80) | SGLT-2i | 0.93 (0.80-1.08) |
|  | **latency = 60 days** | DPP-4i | 1.00 (ref) | DPP-4i | 1.00 (ref) |
|  |  | TZD | 0.67 (0.56-0.79) | SGLT-2i | 0.94 (0.81-1.09) |
|  | **latency = 90 days** | DPP-4i | 1.00 (ref) | DPP-4i | 1.00 (ref) |
|  |  | TZD | 0.69 (0.58-0.81) | SGLT-2i | 0.95 (0.83-1.10) |
|  | **latency = 120 days** | DPP-4i | 1.00 (ref) | DPP-4i | 1.00 (ref) |
|  |  | TZD | 0.70 (0.60-0.82) | SGLT-2i | 0.96 (0.83-1.10) |

Abbreviations: DPP-4i, dipeptidyl peptidase-4 inhibitors; TZD, thiazolidinediones; SGLT-2i, sodium-glucose cotransporter-2 inhibitors; HR, hazard ratio; CI, confidence interval.
