# Supplementary material for: Association between hypoglycemic agent use and the risk of occurrence of nonalcoholic fatty liver disease in patients with type 2 diabetes mellitus
Source: PLoS One. 2023 Nov 22;18(11):e0294423. doi: 10.1371/journal.pone.0294423 (PMC10664876; doi:10.1371/journal.pone.0294423)
Supplement: Attachment — Submitted filename: Response to Comments_Jul21,2023.docx [file pone.0294423.s007.docx]

**Manuscript Number:** **PONE-D-23-12492**

**Title:** **Association between hypoglycemic agent use and the risk of occurrence of nonalcoholic fatty liver disease in patients with type 2 diabetes mellitus**

**Response to Comments**

Journal requirements:

When submitting your revision, we need you to address these additional requirements.

- **Requirement 1:**

Please ensure that your manuscript meets PLOS ONE's style requirements, including those for file naming. The PLOS ONE style templates can be found at <https://journals.plos.org/plosone/s/file?id=wjVg/PLOSOne_formatting_sample_main_body.pdf> and <https://journals.plos.org/plosone/s/file?id=ba62/PLOSOne_formatting_sample_title_authors_affiliations.pdf>

**Response:**

Thank you. We revised the manuscript in accordance to the PLOS ONE’s style requirements:

- Reformatted title page (resized font size, inserted lines, aligned left for author list)
- Reformatted headings in main body (applied Styles)
- Refence based on PLoS EndNote output styles
- Changed file namings according to the guided formats (eg., ‘S1_Fig.tif’, ‘Fig1.tif’)
- Changed the section heading in sentence case
   🡪 ‘Methods and Materials’ 🡪 ‘Methods and materials’ (page 4)
- Changed in text citations for table and figure:
   🡪S2 table 🡪 S2 Table (page 10)
   🡪 Fig 2 and Fig 3 🡪 Figs 2 and 3 (page 10)
- **Requirement 2:**

Please provide additional details regarding participant consent. In the ethics statement in the Methods and online submission information, please ensure that you have specified (1) whether consent was informed and (2) what type you obtained (for instance, written or verbal, and if verbal, how it was documented and witnessed). If your study included minors, state whether you obtained consent from parents or guardians. If the need for consent was waived by the ethics committee, please include this information.

If you are reporting a retrospective study of medical records or archived samples, please ensure that you have discussed whether all data were fully anonymized before you accessed them and/or whether the IRB or ethics committee waived the requirement for informed consent. If patients provided informed written consent to have data from their medical records used in research, please include this information.

**Response:**

Thank you. We ensure that we have already provided the following ethics statement both in the Methods (page 4) and online submission site. This study was waived from the need of informed consent from ethics committee since the data we utilized is a fully anonymized data.

“This study granted a waiver for the need of informed consent from individuals by the Institutional Review Board of Sookmyung Women’s University, Seoul, Republic of Korea, since the NHIS masked personal identifiers to de-identified join keys to secure ethical clearance (No. SMWU-2204-HR-016).”

- **Requirement 3:**

Thank you for stating the following financial disclosure:

“This work was supported by the National Research Foundation of Korea (NRF) grant funded by the Korean government (MSIT) [grant number NRF-2021R1F1A1052976].”

Please state what role the funders took in the study. If the funders had no role, please state: "The funders had no role in study design, data collection and analysis, decision to publish, or preparation of the manuscript."

If this statement is not correct you must amend it as needed.

Please include this amended Role of Funder statement in your cover letter; we will change the online submission form on your behalf.

**Response:**

Thank you for your guidance. We added the statement in the cover letter attached as a separate file.

“We would like to provide further information on financial disclosure by confirming the funder of this study, National Research Foundation of Korea, had no role in study design, data collection and analysis, decision to publish, or preparation of the manuscript.”

”

Reviewer(s)’ Comments to Author:

***Reviewer 1:***

- **Comment 1. Propensity score estimation**

There should be more clarity on how propensity scores were calculated.
<1> page 8, line 175-176: “The propensity score of individuals was calculated by considering covariates through a multivariate logistic regression analysis.”
<2> page 8, line 167-171: “The BMI of each patient was assessed for a period of 1 year prior to and 1 year after the index date…”, “Concomitant medications were defined as medications that individuals take for at least one overlapping day within the follow-up period.”

Do the covariates mentioned in the sentence in <1> include BMI and concomitant medications? If so, based on the statements in <2>, it is concerning that post-index date covariates were included in the model used to calculate the propensity scores. This raises concerns because including post-baseline covariates may introduce biases influenced by treatment. Especially considering that both TZD and SGLT2 inhibitors, which are the drugs of interest in this study, are known to affect body weight when taken.

Therefore, selecting post-treatment BMI as a conditional covariate for calculating the propensity score (the probability of treatment assignment) is not reasonable. Thus, it is necessary to specify the rationale for selecting covariates in propensity score calculation and describe that the propensity scores were calculated using an appropriate model.

**Response:**

*(Please note that referenced lines and pages mentioned herein are based on the clean version, not the tracked version, of the revised manuscript.)* Thank you for your comment. We clarified the propensity score matching method in line 184 (page 8).

“Propensity score matching was performed using nearest neighbor matching at a 1:3 ratio to secure statistical power and minimize the number of dropped samples.”

Regarding BMI, we added descriptions on the rationale of BMI definition and provided additional references along the change in line 174-179 (page 8).

“Baseline BMI of each patient was defined as a single-point value of BMI measured on the closest date from the index date within the 2 years of time window: a period between 1 year prior and after the index date. This definition of baseline BMI was to incorporate as much available data as possible, given that BMI records were only available on an annual or biennial basis in NHIS-NSC database [15, 16].”

Added references are as follows:

15. Lee J, Lee JS, Park S-H, Shin SA, Kim K. Cohort Profile: The National Health Insurance Service–National Sample Cohort (NHIS-NSC), South Korea. International Journal of Epidemiology. 2016;46(2):e15-e. doi: 10.1093/ije/dyv319.

16.Song DK, Hong YS, Sung Y-A, Lee H. Body mass index and stroke risk among patients with diabetes mellitus in Korea. Plos one. 2022;17(9):e0275393.

Also, we addressed the related limitations in line 422-425 (page 20).

“Lastly, the findings of this study should be interpreted with caution considering the possible bias despite the application of various statistical measures. For instance, including BMI or comedication information that have been captured during the follow up period may have introduced post-treatment bias, …”

- **Comment 2. Outcome definition-1**

It is highly likely that the variability in the prevalence of NAFLD across studies, as mentioned by the authors on “page 16, lines 310-317”, is due to the lack of a valid definition for NAFLD. In this study, NAFLD was defined as K76.0 and K75.8.

🡪 Has there been a validation study conducted to define NAFLD using diagnostic codes in the NHIS sample cohort? If such a study exists, it would be advisable to mention it. There is a need to strengthen the description of the validity of the outcome.

**Response:**

Thank you for your comment. We added a following description and supporting references regarding the definition for NAFLD in line 409-416 (page 19-20).

“Firstly, regarding the definition of NAFLD, algorithms would have been more precise to incorporate laboratory findings or imaging or biopsy data [35]. However, defining NAFLD based on disease codes is considered a feasible option when data source lacks clinical information [36]. Although validation of the diagnosis code-based definition was limited within NHIS sample cohort data, previous population-based studies have reported an accuracy of 85% with utilizing diagnostic codes for defining NAFLD [37]. Nevertheless, it is important to acknowledge that this study may not reflected the real disease status owing to the nature of claims data.”

Added references are as follows:

35. Corey KE, Kartoun U, Zheng H, Shaw SY. Development and Validation of an Algorithm to Identify Nonalcoholic Fatty Liver Disease in the Electronic Medical Record. Dig Dis Sci. 2016;61(3):913-9. Epub 2015/11/06. doi: 10.1007/s10620-015-3952-x. PubMed PMID: 26537487; PubMed Central PMCID: PMCPMC4761309.

36.	Terai S, Buchanan-Hughes A, Ng A, Lee IH, Hasegawa K. Comorbidities and healthcare costs and resource use of patients with nonalcoholic fatty liver disease (NAFLD) and nonalcoholic steatohepatitis (NASH) in the Japan medical data vision database. Journal of Gastroenterology. 2021;56(3):274-84. doi: 10.1007/s00535-021-01759-2.

37.	Allen AM, Therneau TM, Larson JJ, Coward A, Somers VK, Kamath PS. Nonalcoholic fatty liver disease incidence and impact on metabolic burden and death: A 20 year-community study. Hepatology. 2018;67(5):1726-36. Epub 2017/09/25. doi: 10.1002/hep.29546. PubMed PMID: 28941364; PubMed Central PMCID: PMCPMC5866219.

- **Comment 2. Outcome definition-2**

Alternatively, sensitivity analysis should be performed to validate the robustness of the outcome definition.

**Response:**

Thank you for your comment. However, we regret to mention that we were unable to conduct additional analyses for the suggested revision due to the limited period of data accessibility allowed by NHIS. Thus, we mentioned an importance of further research to validate our findings with more rigorous statistical analysis as a part of study limitations in line 426-428 (page 20).

“Thus, while this study offers valuable insights into NAFLD management in patients with type 2 diabetes, further research that validate these findings through rigorous statistical approach are needed.”

- **Comment 3. Sensitivity analysis-1**

There are some unclear aspects regarding the sensitivity analysis that applied alternative criteria to reassign the treatment groups. It is necessary to provide clearer descriptions in this regard.
<1> Page 9, line 190-192: “…, alternative criterion assigned patients to groups based on the drug they used for the longest period during the follow-up period.”
<2> Page 18, line 361-363: “Interestingly, the proportion of SGLT-2i users in Cohort 2 increased slightly from 13.3% in main analysis to 14.6% in sensitivity analysis, which indicates that some patients may have switched from DPP-4i to SGLT-2i and used SGLT-2 inhibitors for longer periods than DPP-4i.”

According to the statements in <1> and <2>, if a “patient A” initially used DPP-4i and later switched to SGLT-2i, and the duration of SGLT-2i use was longer than the duration of DPP-4i use, then this patient would be considered as part of the SGLT-2i group when calculating the hazard ratio (HR).

🡪 If “patient A” switches from DPP4i to SGLT2i, is the person-time calculated using the new index date as the date “patient A” starts SGLT2i? Then, what about the person-time prior to the switch, i.e. while taking DPP4i? Did you include that in the person-time for the DPP4i group or ignore it?

**Response:**

Thank you for your comment. Yes, the person-time prior to switch in “patient A” was not considered when calculating the hazard ratio (HR) in sensitivity analysis. The alternative definition for drug exposure was applied to grouping the patients into each drug class, and otherwise, the methods were identical with the main analysis (e.g., further drug exposure was assessed with as-treated approach; study timeframes for exclusion, covariate assessment, and outcome observation were all set based on the index date).

- **Comment 3. Sensitivity analysis-2**

🡪 If “patient A” experience an outcome while on DPP-4i, is this patient censored, or will he/she be assigned to the SGLT2i group (in the future) because they have been on SGLT2i for longer, so the outcome before they are assigned to SGLT-2i is not considered?

**Response:**

Thank you for your comment. Yes, outcome prior to the new index date, or the first prescription date of SGLT-2i in “patient A” case, was not considered. However, if “patient A” had a history of NAFLD prior to the new index date, he/she would not be included as the eligible cohort. In other words, patient A would not be given a chance to be followed-up for the outcome. This is due to the study setting where both the definition of the new index date and patient exclusion came after assigning patients to either the control or comparative group. Therefore, the possibility of immortal time bias could be considered neglectable in this sensitivity analysis, as not only the patient-time prior to the switch to SGLT-2i, but also the outcome before the new index date were excluded from the eligible cases in the survival analysis.

***Reviewer #2:***

- **Overall comment:**

This retrospective cohort study examined the risk of nonalcoholic fatty liver disease (NAFLD) in patients with type 2 diabetes using different hypoglycemic agents. The study utilized data from the National Health Insurance Service–National Sample Cohort of South Korea. The findings showed that the use of thiazolidinediones (TZD) was associated with a reduced risk of NAFLD compared to dipeptidyl peptidase-4 inhibitors (DPP-4i). Sodium-glucose cotransporter-2 inhibitors (SGLT-2i) also demonstrated a lower risk of NAFLD, although not statistically significant. Sensitivity analysis considering the longer duration of drug use further supported the protective effect of SGLT-2i on NAFLD. These results suggest that prioritizing TZD and SGLT-2i over DPP-4i may help decrease the risk of NAFLD in patients with type 2 diabetes.

However, there are several major points that need to be addressed. In particular, the method for determining the NAFLD outcome needs to be revised.

- **Comment 1:**

Due to the low diagnostic validity of NAFLD, it is not possible to capture the outcome using ICD codes. To determine the outcome, validity data for the outcome or algorithms such as those described in the following reference should be applied:

*Pradhan R, Yin H, Yu O, Azoulay L. Glucagon-Like Peptide 1 Receptor Agonists and Sodium-Glucose Cotransporter 2 Inhibitors and Risk of Nonalcoholic Fatty Liver Disease Among Patients With Type 2 Diabetes. Diabetes Care. 2022 Apr 1;45(4):819-829. doi: 10.2337/dc21-1953. PMID: 35104330.*

**Response:**

Thank you for your comment. We added a following description and supporting references regarding the definition for NAFLD in line 409-416 (page 19-20).

“Firstly, regarding the definition of NAFLD, algorithms would have been more precise to incorporate laboratory findings or imaging or biopsy data [35]. However, defining NAFLD based on disease codes is considered a feasible option when data source lacks clinical information [36]. Although validation of the diagnosis code-based definition was limited within NHIS sample cohort data, previous population-based studies have reported an accuracy of 85% with utilizing diagnostic codes for defining NAFLD [37]. Nevertheless, it is important to acknowledge that this study may not reflected the real disease status owing to the nature of claims data.”

Added references are as follows:

35. Corey KE, Kartoun U, Zheng H, Shaw SY. Development and Validation of an Algorithm to Identify Nonalcoholic Fatty Liver Disease in the Electronic Medical Record. Dig Dis Sci. 2016;61(3):913-9. Epub 2015/11/06. doi: 10.1007/s10620-015-3952-x. PubMed PMID: 26537487; PubMed Central PMCID: PMCPMC4761309.

36.	Terai S, Buchanan-Hughes A, Ng A, Lee IH, Hasegawa K. Comorbidities and healthcare costs and resource use of patients with nonalcoholic fatty liver disease (NAFLD) and nonalcoholic steatohepatitis (NASH) in the Japan medical data vision database. Journal of Gastroenterology. 2021;56(3):274-84. doi: 10.1007/s00535-021-01759-2.

37.	Allen AM, Therneau TM, Larson JJ, Coward A, Somers VK, Kamath PS. Nonalcoholic fatty liver disease incidence and impact on metabolic burden and death: A 20 year-community study. Hepatology. 2018;67(5):1726-36. Epub 2017/09/25. doi: 10.1002/hep.29546. PubMed PMID: 28941364; PubMed Central PMCID: PMCPMC5866219.

- **Comment 2:**

If exclusions are made after the follow-up period following propensity score matching, the balance achieved by the propensity score matching may be compromised. To incorporate adherence, it is recommended to utilize indicators such as PDC, MPR, and perform subgroup analyses.

**Response:**

Thank you for your comment. We added a description regarding a possible bias as a limitation in line 423-426 (page 20).

“Lastly, the findings of this study should be interpreted with caution considering the possible bias despite the application of various statistical measures. For instance, …, and excluding non-adherent patients by assessing 30 days after the index date may potentially led to selection bias.”

We agree with utilizing indicators for adherence, but unfortunately, conducting additional analysis was unavailable due to the limited period of data accessibility allowed by NHIS. Thus, we added following sentence as a study limitation in line 426-428 (page 20).

“Thus, while this study offers valuable insights into NAFLD management in patients with type 2 diabetes, further research that validate these findings through rigorous statistical approach are needed.”

- **Comment 3:**

There are no references provided for key claims (e.g., the neutral effect of DPP-4i on liver fat or the definition of the outcome).

**Response:**

Thank you for your comment. We added the following reference to the claim in line 91 (page 4).

“New-users of DPP-4i were set as an active comparator, since DPP-4i has been known to have a neutral effect on body weight and liver fat, and also because it is considered a therapeutic alternative to TZD or SGLT-2i as a second-line drug for type 2 diabetes [14]”

Citation of added reference is as follows:

14. Dougherty JA, Guirguis E, Thornby K-A. A systematic review of newer antidiabetic agents in the treatment of nonalcoholic fatty liver disease. Annals of Pharmacotherapy. 2021;55(1):65-79.

- **Comment 4:**

The exposure assessment method is not clearly described. It seems to have adopted an as-treated approach, but the description needs to be clarified.

**Response:**

Thank you for your comment. We added a description regarding the exposure assessment in line 134-138, page 6)

“Drug exposure was assessed based on as-treated approach to consider only the person-time when patients are on the initially assigned treatment is evaluated. Accordingly, patients were followed up from the index date and were censored at the earliest date among when deviation from the initial exposure happened (discontinuation of the initial drug or addition of the other study drug), end of study date, or death.”

- **Comment 5:**

The median follow-up for TZD and SGLT2i is significantly lower than that for DPP-4i in relation to the outcome of NASH. The reason for this discrepancy should be addressed.

**Response:**

Thank you for your comment. We added descriptions in discussion and supporting references:

(line 339-342, page 16): for comparison between DPP-4i and TZD;

“The median follow-up durations were 1.4 and 0.8 years for DPP-4i and TZD, respectively, and this discrepancy in the follow-up period was also found in previous studies. It can be explained by relatively better tolerance of DPP-4i compared to TZD, which may have resulted in the higher persistency in DPP-4i group than in TZD group [23].”

Citation for added reference is as follows:

23. Gor D, Lee TA, Schumock GT, Walton SM, Gerber BS, Nutescu EA, et al. Adherence and persistence with DPP-4 inhibitors versus pioglitazone in type 2 diabetes patients with chronic kidney disease: a retrospective claims database analysis. Journal of Managed Care & Specialty Pharmacy. 2020;26(1):67-75.

(line 370-373, page 18): for comparison between DPP-4i and SGLT-2i.

“Median follow-up durations were longer in DPP-4i compared to that of SGLT-2i. Similar to the discrepancy in the follow-up period in Cohort 1, this is speculated as the result that reflects poor adherence of SGLT-2i [32].”

Citation for added reference is as follows:

32. Ofori‐Asenso R, Sahle BW, Chin KL, Mazidi M, Ademi Z, De Bruin ML, et al. Poor adherence and persistence to sodium glucose co‐transporter 2 inhibitors in real‐world settings: Evidence from a systematic review and meta‐analysis. Diabetes/Metabolism Research and Reviews. 2021;37(1):e3350.

- **Comment 6:**

The point estimate of SGLT2i shows different trends in certain subgroups. The reason for this discrepancy should be discussed.

**Response:**

Thank you for your comment. We added in discussion and supporting reference section (line 378-386, page 18).

“When analyzed by subgroups within Cohort 2, the results were mostly in line with the main analysis except in those who used ipragliflozin (HR, 1.21; 95% CI: 0.63-2.33) and in those with BMI <23 kg/m^2^ (HR, 1.11; 95% CI: 0.44-0.99), but with no statistical significance. Relatively small number of patients who used ipragliflozin compared to the other SGLT-2i drugs may be attributed to this result (e.g., ipragliflozin users (n=88) vs. dapagliflozin users (n=1075)), or from a distinct phenotype of NAFLD in those with BMI <23 kg/m^2^, who are referred as ‘lean NAFLD’, since these patients are known to have lower metabolic risk but higher fibrosis score and cardiovascular risk than NAFLD within other BMI groups [33].”

Citation for added reference is as follows:

33. Xu R, Pan J, Zhou W, Ji G, Dang Y. Recent advances in lean NAFLD. Biomedicine & Pharmacotherapy. 2022;153:113331.

- **Comment 7:**

The flow chart in Figure 1 may be misleading to readers, especially regarding the process of constructing cohort 1 and cohort 2. Comments explaining this process should be provided.

**Response:**

Thank you. I made amendments to Figure 1 to clarify the process of constructing the cohorts. Also, we added a more detailed description in the manuscript (line 207-212, page 9)

“A total of 65,224 patients were grouped into each group, in a non-mutually exclusive manner, of 50,094, 19,571, and 11,127 patients with a history of using DPP-4i, TZD, and SGLT-2i, respectively. Subsequentially, two groups of patients with corresponding comparator and control drugs were grouped as the sum of sets to construct Cohort 1 and Cohort 2 (e.g., Cohort 1 included those prescribed with DPP-4i or TZD at least once in 2003 - 2019).”

- **Comment 8:**

It is recommended to conduct sensitivity analyses in various ways. This includes not only intention-to-treat analysis but also exploring other PS techniques such as outcome definition modification or IPTW to measure average treatment effects among the whole population.

**Response**:

Thank you for your comment. As suggested, we believe that application of further statistical approach would enhance robustness of our results. However, additional analysis was unavailable due to the limited period of data accessibility allowed by NHIS. Thus, we mentioned an importance of further research to validate our findings in line 426-428 (page 20)

“Thus, while this study offers valuable insights into NAFLD management in patients with type 2 diabetes, further research that validate these findings through rigorous statistical approach are needed.”

- **Comment 9:** Line 386: NASH (75.8) should be corrected to NASH (K75.8).

**Response**:

Thank you for your correction. Revised as accordingly, but we decided to delete the corresponding line during the revision.
