# Supplementary material for: Association between hypoglycemic agent use and the risk of occurrence of nonalcoholic fatty liver disease in patients with type 2 diabetes mellitus
Source: PLoS One. 2023 Nov 22;18(11):e0294423. doi: 10.1371/journal.pone.0294423 (PMC10664876; doi:10.1371/journal.pone.0294423)
Supplement: Attachment — Submitted filename: Response to Comments_13Sep2023.docx [file pone.0294423.s008.docx]

**Manuscript Number:** **PONE-D-23-12492**

**Title:** **Association between hypoglycemic agent use and the risk of occurrence of nonalcoholic fatty liver disease in patients with type 2 diabetes mellitus**

**Response to Comments**

***Reviewer #1***

- **Comment 1:**

If patients who switched from DPP4i to SGLT2i were not taken into account for person-time during their DPP4i usage period when calculating the hazard ratio, there is a possibility that the relative risk derived in this manner may overestimate the preventive effects of SGLT2i.

Furthermore, if individuals who experienced the outcome while using other medications before the new-index date were excluded, it implies that susceptible individuals were depleted, leading to potential selection bias.

Considering that this sensitivity analysis may cause confusion among readers, I think it’s better to leave it out.

- **Response:**

Thank you for your valuable comment. I agree with your concerns regarding possible time-related biases. Although the sensitivity analysis using alternative definition for a drug exposure has been applied to consider prescription practice ─frequent switching in different oral antidiabetic agents especially for the initial treatment for type 2 diabetes─ into the exposure definition, I have left the relevant parts out, as suggested. I believe that this revision has brought more clarity in terms of study design and improved delivery.

- *Deleted line 44-46 (tracked file), page 2*
- *Deleted line 206-210 (tracked file), page 9*
- *Deleted line 320-323 (tracked file), page 15*
- *Deleted line 386-391 (tracked file), page 18*
- *Deleted line 399-402, line 406-7 (tracked file), page 19*

***Reviewer #2***

**Comment 1 & 3:**

- **Comment 1:** To my knowledge NHIS-NSC data source they used provides the same format of data within 5 months upon request. Additionally, it is known to support storage for further analysis. I recommend authors to conduct additional analysis.
- **Comment 3**: Please perform intention-to-treat analysis for the sensitivity analysis.

**Response:**

- Thank you for your recommendation. We acknowledge your suggestion that our study could benefit from additional analysis. However, NHIS-NSC do provide data storage services are only available for requests made prior to the data expiration, which was not applicable to our case. While additional analysis wasn’t feasible, we believe that our manuscript still contributes meaningfully to the expanding body of literature on antidiabetic drugs and NAFLD.

**Comment 2:**

- The current sensitivity analysis appears to be an analysis of prevalent users. Please clarify and describe what is meant by "prevalent user" in this context.

**Response:**

- We conducted sensitivity analysis for varying latency periods within the PS-matched study population, comprised of newly diagnosed type 2 diabetes who were also new user of each antidiabetic medication. Regarding the term ‘prevalent users’, I am concerned that I may have misunderstood, but to my understanding, this term has not been used in our manuscript, nor in our previous response to comments.
- Additionally, it is important to note that we omitted another sensitivity analysis for an alternative definition of drug exposure based on the other reviewer’s suggestion.

**Comment 4:**

- I have already suggested ways to improve outcome validity (e.g., diabetes care). Or authors might be possible to utilize the Fatty Liver Index or Hepatic Steatosis Index. It should be noted that using ICD-10 for analysis may lead to significant underestimation of NAFLD and NASH and has not been validated in the Korean population. Therefore, addressing this issue is essential.

**Response:**

- Thank you for your comment. We understand your concern and we had considered incorporating additional definition tools like FLI or HSI. However, the limited clinical availability of clinical variables such as triglycerides or liver function test readings in NHIS-NSC made it impossible to apply these tools. Moreover, the inclusion of medication use was also not feasible since there are currently no reimbursed medications indicated for NAFLD.
- Therefore, using the diagnosis code alone to define the NAFLD was the last option possible, which is also addressed as an alternative measure in the previous study. (Terai S et al., 2021). For this revision, we elaborated more in the discussion section on study limitation regarding the NAFLD definition (*Line 404-411 (clean file), page 19*):
  - “Despite these strengths, this study has several limitations. Firstly, owing to the limited clinical information in NHIS database, we used ICD-10 codes to define the occurrence of NAFLD, which may have led to underestimation of the outcome [35]. Although defining NAFLD using disease codes is considered as an alternative option when data source lacks clinical information given the reported accuracy of 85% for defining NAFLD, its accuracy within the Korean population has not been fully validated [36, 37]. Therefore, further studies that incorporate clinical variables such as NAFLD prediction index or imaging data are needed to validate the current findings.”

**Comment 5:**

- Please provide a description of how missing values were handled in the BMI data.

**Response:**

- Thank you for your comment. In this study, no imputation has been made for missing data for BMI in the original data source. We added the sentence, “Observations with missing values in the BMI data were excluded from the analysis.” (*Line 186-7 (clean file), page 8*). Also, we added the count of missing values in BMI within each cohort as a footnote under the Table 3 (*Line 244 (clean file), page 11)* and Table 4 *(Line 260 (clean file), page 13).*
